# Neuroprotective Effects of Huperzine B and *Huperzia carinata* Extract Against MPP^+^‐Induced Oxidative Stress in SH‐SY5Y Cells

**DOI:** 10.1155/adpp/6446024

**Published:** 2026-09-21

**Authors:** Kittikun Viwatpinyo, Poommaree Namchaiw, Pipob Suwanchaikasem, Sujira Mukda, Sudichhya Tamrakar, Amit Jaisi, Sakan Warinhomhoun

**Affiliations:** ^1^ School of Medicine, Walailak University, Thai Buri Nakhon Si Thammarat, 80160, Thailand, wu.ac.th; ^2^ Herbology Research Center, Walailak University, Thai Buri Nakhon Si Thammarat, 80160, Thailand, wu.ac.th; ^3^ Biological Engineering Program, Faculty of Engineering, King Mongkut’s University of Technology Thonburi, Bangkok, Thailand, kmutt.ac.th; ^4^ Neuroscience Center for Research and Innovation, Learning Institute, King Mongkut’s University of Technology Thonburi, Bangkok, Thailand, kmutt.ac.th; ^5^ Baiya Phytopharm Co., Ltd., Chulalongkorn University, Bangkok 10330, Thailand, chula.ac.th; ^6^ Research Center for Neuroscience, Institute of Molecular Biosciences, Mahidol University, Nakorn Pathom 73170, Thailand, mahidol.ac.th; ^7^ School of Pharmacy, Walailak University, Thasala Nakhon Si Thammarat, 80160, Thailand, wu.ac.th; ^8^ Department of Chemistry, Faculty of Sciences, Chulalongkorn University, Bangkok 10330, Thailand, chula.ac.th; ^9^ College of Oriental Medicine, Rangsit University, Pathum Thani 12000, Thailand, rsu.ac.th

**Keywords:** *Huperzia carinata*, huperzine B, oxidative stress, Parkinson’s disease, SH-SY5Y cells

## Abstract

Parkinson’s disease (PD) is a neurodegenerative disorder associated with dopaminergic neuronal loss, mitochondrial dysfunction, and redox imbalance. Huperzine B (HupB), an alkaloid derived from *Huperzia* species, has demonstrated antioxidant and acetylcholinesterase‐inhibitory activity; however, its effects in toxin‐induced cellular models of mitochondrial oxidative injury remain incompletely characterized. This study investigated the effects of HupB and an extract derived from *Huperzia carinata* (E1) in 1‐methyl‐4‐phenylpyridinium (MPP^+^)‐induced SH‐SY5Y cells. LC–MS/MS analysis identified Huperzines A, B, and caffeic acid in E1. Cells were pretreated with HupB or E1 prior to exposure to 1 mM MPP^+^. Cell viability, nuclear morphology, reactive oxygen species (ROS), antioxidant enzyme activity, mitochondrial membrane potential (JC‐1), apoptosis‐related proteins (Bax/Bcl‐2), and global proteomic profiles were evaluated. HupB and E1 attenuated MPP^+^‐induced reductions in cell viability and ROS elevation, while partially improving glutathione levels and catalase and superoxide dismutase activities. MPP^+^ significantly decreased mitochondrial membrane potential, whereas pretreatments did not produce significant restoration. Similarly, Bax/Bcl‐2 expression showed no significant modulation despite trends toward normalization at higher concentrations. Proteomic analysis revealed altered expression of proteins associated with negative regulation of apoptosis and cellular stress responses. These findings indicate that HupB and *H. carinata* extract are associated with modulation of oxidative and proteostasis‐related responses in an MPP^+^‐induced undifferentiated SH‐SY5Y cellular injury model, supporting further investigation in more physiologically relevant systems.

## 1. Introduction

Parkinson’s disease (PD) is a progressive neurodegenerative disorder characterized by the loss of dopaminergic neurons in the substantia nigra pars compacta of the midbrain, which predominantly affects movement control. It is characterized by distinctive motor symptoms, including bradykinesia, rigidity, resting tremor, and postural instability, as well as a range of nonmotor symptoms, such as cognitive impairment, depression, and autonomic dysfunction, which are associated with dopamine deficits. Beyond the dopaminergic system, PD affects other neurotransmitter pathways, including cholinergic, serotonergic, and noradrenergic networks, which might contribute to psychiatric symptoms. PD affects approximately 1%–2% of the population who are > 60 years of age, and its incidence is predicted to considerably rise as life expectancy increases [1]. The accumulation of misfolded alpha‐synuclein in Lewy bodies is a hallmark pathological feature that contributes to neurodegeneration through mitochondrial dysfunction, oxidative stress, and neuroinflammation [2, 3]. Although mutations in the *SNCA* gene, which encodes alpha‐synuclein, account for certain familial cases of PD, genome‐wide association studies have identified numerous risk loci associated with idiopathic PD. Moreover, the pathophysiology of PD is suggested to involve a complex relationship between genetic predisposition, epigenetic alterations, and environmental factors, such as iron accumulation and exposure to pesticides and insecticides [4–6]. Current treatments for PD are largely symptomatic, where dopamine replacement therapy with levodopa remains the gold standard [7]. However, long‐term levodopa use is associated with motor complications, including dyskinesia, early morning akinesia, and unpredictable off periods [8, 9]. Alternative pharmacological approaches, such as the use of dopamine agonists, monoamine oxidase inhibitors, and catechol‐*O*‐methyltransferase inhibitors, provide additional symptom relief [2, 7]. Furthermore, alleviating mitochondrial dysfunction is expected to be a novel approach to compensate for neuronal bioenergetic defects and thereby slow the progression of PD [10].

The use of herbal medicines from plant sources as alternative or adjunct therapies for PD has received increasing interest due to their neuroprotective properties, and numerous herbal medicines have been investigated for their potential protective effects against PD [11]. Velvet bean (*Mucuna pruriens*), a natural source of levodopa, has demonstrated efficacy in alleviating PD symptoms by increasing dopamine levels while minimizing dyskinesia effects [12, 13], capable of reducing oxidative injury in rat brains [14], and its seed extract has been reported to protect against dopaminergic neuronal loss and behavioral impairment in MPTP‐treated mice [15]. Chlorogenic acid, a natural polyphenol compound found in coffee beans and berries, has been shown to attenuate mitochondrial dysfunction and dopaminergic neuronal apoptosis in MPTP‐intoxicated mice, partly through modulation of oxidative stress and intrinsic apoptotic signaling pathways [16]. Similarly, ursolic acid found in fruit peels reduced neuroinflammation and oxidative damage in MPTP‐induced models and improved motor deficits in association with preservation of nigrostriatal integrity [17, 18]. Curcumin, derived from *Curcuma longa*, restores depleted glutathione (GSH) levels, protects against oxidative injury, maintains mitochondrial Complex I activity, which is typically impaired as a result of GSH loss [19], and offers elevated protection against peroxynitrite‐mediated injury in dopaminergic neuronal cultures [20]. Moreover, a root extract from *Withania somnifera* (Ashwagandha) has been shown to improve neuronal functions in a mouse model of PD, which included increases in superoxide dismutase (SOD) and catalase (CAT) activities [21], recovery of catecholamine levels, and improvement in motor functions [22]. In paraquat–maneb‐induced and MPTP‐related mouse models, *W*. *somnifera* alleviated dopaminergic neuronal loss and improved motor deficits, partly through suppression of apoptotic signaling pathways and attenuation of oxidative stress [23, 24]. Extracts from *Ginkgo biloba* have also been studied for their antioxidative properties and were revealed to be capable of restoring the levels of CAT, SOD, and GSH‐related enzymes in the brain of a rat model of PD [25]. Recently, *Aesculus hippocastanum* extract was shown to exert protective effects in an 1‐methyl‐4‐phenylpyridinium (MPP^+^) model through the activation of peroxisome proliferator‐activated receptor gamma (PPARγ), implicating anti‐inflammatory and transcriptional regulatory pathways in neuronal survival [26]. Marine‐derived tetramic acid‐motif compounds isolated from *Tolypocladium cylindrosporum* also demonstrated anti‐Parkinson activities in cellular models, highlighting the therapeutic potential of structurally diverse natural metabolites [27]. Similarly, *Artemisia* leaf extract has been reported to attenuate MPP^+^/MPTP‐induced neurotoxicity by activating TRPML1 and promoting autophagy–mitophagy clearance mechanisms in both in vitro and in vivo systems [28]. Collectively, these findings underscore the growing interest in plant‐ and marine‐derived compounds as modulators of toxin‐induced mitochondrial stress and proteostatic imbalance. However, despite accumulating evidence of cytoprotective effects, comprehensive characterization of global proteomic alterations induced by such compounds in MPP^+^‐based models remains limited.

The herbal extract of interest in our study was Huperzine B (HupB), a bioactive alkaloid primarily found in the *Huperzia* genus [29]. This compound is structurally related to HupA, a natural alkaloid being investigated for reducing symptoms of Alzheimer’s disease. HupB has also been identified in related species within the *Huperzia selago* complex, which exhibits significant variability in alkaloid content [30]. HupB exhibits beneficial effects toward neurons through several mechanisms. One of its primary actions is the inhibition of acetylcholinesterase (AChE), an enzyme that breaks down acetylcholine, a crucial neurotransmitter for cognitive function. This inhibitory effect may enhance cholinergic transmission and improve memory and learning. In addition to its role in AChE inhibition, studies have indicated that HupB can protect PC12 rat pheochromocytoma cells from hydrogen peroxide‐induced damage by increasing antioxidant enzyme activity, reducing lipid peroxidation [31], and reducing PC12 cell injury after exposure to oxygen–glucose deprivation, likely by increasing SOD enzyme activity [32]. Although the antioxidant and AChE‐inhibitory properties of HupB have been previously reported, its effects in cellular models relevant to PD‐associated mitochondrial oxidative stress remain insufficiently characterized. In particular, little is known about how HupB influences global protein expression profiles under conditions of mitochondrial stress induced by MPP^+^, a widely used Complex I inhibitor that mimics PD‐related oxidative injury. Moreover, while extracts from *Huperzia* species contain multiple bioactive alkaloids and phenolic compounds, comparative evaluation of purified HupB and crude *Huperzia carinata* extracts in the same MPP^+^‐induced SH‐SY5Y oxidative injury model has not been systematically performed. Since MPP^+^ selectively inhibits mitochondrial Complex I and leads to the accumulation of oxygen radicals, it provides a suitable platform to investigate redox‐modulating natural compounds, although it does not recapitulate the full spectrum of PD‐specific molecular pathology.

SH‐SY5Y human neuroblastoma cells are widely employed in PD research due to their catecholaminergic characteristics and susceptibility to mitochondrial toxins such as MPP^+^. A systematic review demonstrated that SH‐SY5Y cells represent one of the most frequently used in vitro models in PD‐related studies, particularly for investigating mitochondrial dysfunction, oxidative stress, and apoptosis‐associated mechanisms [33]. Furthermore, several studies have utilized MPP^+^‐induced SH‐SY5Y injury to evaluate neuroprotective compounds targeting redox imbalance and mitochondrial stress pathways [34, 35]. Although undifferentiated SH‐SY5Y cells do not fully recapitulate the phenotype of mature dopaminergic neurons, they provide a reproducible and mechanistically relevant platform for studying mitochondrial Complex I inhibition and oxidative injury, which are central features of PD pathogenesis. In the present study, dopaminergic marker expression, including tyrosine hydroxylase (TH) and dopamine transporter (DAT), was not assessed; therefore, this model should be interpreted as an undifferentiated SH‐SY5Y mitochondrial oxidative injury model rather than a fully characterized dopaminergic neuronal model.

Therefore, the present study aimed to assess the cytoprotective and antioxidant effects of HupB and an extract from *H. carinata* (E1) in an undifferentiated SH‐SY5Y model of MPP^+^‐induced mitochondrial oxidative injury and to characterize their impact on global proteomic alterations using LC–MS/MS‐based analysis. By identifying differentially expressed proteins (DEPs) associated with apoptotic regulation, cytoskeletal stability, and endoplasmic reticulum protein folding, this study provides exploratory proteomic information beyond conventional oxidative stress assays and offers a comparative evaluation of pure and extract‐derived interventions.

## 2. Materials and Methods

### 2.1. Cell Culture and Reagents

The human neuroblastoma cell line SH‐SY5Y (CRL‐2266), which is commonly used in MPP^+^‐based models of mitochondrial dysfunction and oxidative stress relevant to PD research [33, 34], was obtained from ATCC and maintained in Dulbecco’s modified Eagle’s medium (30‐2002; ATCC) supplemented with 10% fetal bovine serum (F7524; Sigma‐Aldrich, St. Louis, MO, USA) and 10 mL/L penicillin/streptomycin (P4333; Sigma‐Aldrich). Cultures were incubated in a humidified incubator with 5% CO_2_ at 37°C, and the culture media were refreshed every 2 days. Cells were used in an undifferentiated state, and dopaminergic neuronal marker expression was not assessed in the present study. The iodide salt of the neurotoxin, MPP^+^, was purchased from Sigma‐Aldrich (D048), dissolved in ultrapure sterile water to obtain a concentration of 100 mM, and stored at −20°C in the dark until use. Huperzine B (HupB) was purchased from a commercial source (B21167, Yuanve Bio‐Technology). HupB and E1 were dissolved in dimethyl sulfoxide (DMSO) to obtain concentrations of 20 mM and 10 mg/mL, respectively, and stored at −20°C in the dark until use.

### 2.2. Extraction of E1

The *H. carinata* (Desv. Ex Poir.) Trevis plants were purchased from the Potsadet Nursery in Nakhon Si Thammarat, Thailand. The plants were maintained in a greenhouse at the Walailak Botanical Park (Nakhon Si Thammarat, Thailand), and a voucher specimen was deposited at the herbarium of the Walailak Botanical Park (WUNST072024HC). Aerial parts of the plants were washed in tap water and oven‐dried for 36 h at 50°C. The dried plant material was crushed into a fine powder and used for compound extraction via the method described by Yang et al. [36], with slight modifications. Briefly, 100 mg of the dried, powdered plant material was prepared for extraction using 2 mL of 3% aqueous tartaric acid. The sample was sonicated for 30 min at room temperature, followed by centrifugation at 2500 rpm. The upper phase was carefully transferred to a new tube, and this process was repeated three times. The collected E1 extracts were then freeze‐dried and stored at −20°C until further use.

### 2.3. E1 and HupB Metabolite Identification

#### 2.3.1. Sample Preparation

E1 and HupB extracts were dissolved in 100% methanol containing 25 ng/mL sulfadimethoxine (internal standard). Samples were diluted to 5 mg/mL and centrifuged at 14,000 rpm for 10 min. Supernatant was transferred to an LC–MS vial and subjected to LC–MS analysis.

#### 2.3.2. LC–MS/MS Acquisition of the E1 Extract

For metabolite profiling, all samples were analyzed using an Agilent 1290 Infinity II liquid chromatography system coupled to a 6545XT quadrupole time‐of‐flight (QTOF) mass spectrometer (Agilent Technologies, USA). Electrospray ionization (ESI) served as the ion source. Chromatographic separation was performed on an Agilent Poroshell 120 EC‐C18 column (2.1 × 100 mm, 2.7 μm particle size), maintained at 50°C. A sample volume of 10 μL was injected for both positive and negative ionization modes.

The mobile phase consisted of Solvent A (0.1% formic acid in water) and Solvent B (acetonitrile). The gradient program was as follows: 0% B for 0.5 min; increased to 55% B over 10 min; ramped to 75% B in 2 min; to 100% B in 1.5 min; held at 100% B for 3 min; returned to 0% B in 0.5 min; and equilibrated at 0% B for 2.5 min. The flow rate was maintained at 0.4 mL/min. A 10% isopropanol‐in‐water solution was used to wash the needle and seals.

Mass spectrometry data were acquired with an MS1 scan range of 100–1700 m/z and an MS2 scan range of 25–1000 m/z. Key MS parameters were as follows: gas temperature, 325°C; nebulizer pressure, 45 psi; drying gas flow, 13 L/min; sheath gas temperature, 275°C; and sheath gas flow, 12 L/min. The nozzle voltage was set at 500 V, fragmentor at 175 V, and skimmer at 65 V. Capillary voltage was adjusted to 4000 V for positive mode and 3000 V for negative mode. The acquisition rate was 3.35 spectra per second. A maximum of 10 precursor ions per cycle were selected for MS/MS analysis, with a minimum intensity threshold of 5000 counts. Collision energies were set to 20 and 10 eV for positive and negative modes, respectively. Reference ions included purine, trifluoroacetic acid ammonium salt, and hexakis(1H,1H,3H‐tetrafluoropropoxy)phosphazene. All data were acquired in centroid mode.

#### 2.3.3. LC–MS Data Analysis

Raw Agilent .d data were converted to an .abf file using Reifycs Abf converter and uploaded to the MS‐DIAL software. Peak detection, sample alignment, and compound identification were performed under default settings. Reference masses were excluded. Online databases, including ESI (±) MS/MS from authentic standards, Fiehn/Vaniya natural product, and BMDMS‐NP, were used as a metabolite library. Normalization was applied against the sulfadimethoxine internal standard. M + H and M‐H were selected as adducts for positive and negative modes, respectively. Features identified with a sample/blank ratio less than 10 and an identification score < 0.60 were excluded.

### 2.4. Treatment Procedure

In all experiments, the SH‐SY5Y cells were seeded in containers at the appropriate densities and maintained for 24 h. The culture medium was then replaced with various concentrations of HupB or E1 and incubated for 2 h. The concentration ranges of HupB (1–20 μM) and E1 extract (0.1–10 μg/mL) were selected based on preliminary cytotoxicity screening to ensure the absence of intrinsic toxicity and maintenance of cell viability above 90% under basal conditions. These concentrations were chosen to allow evaluation of protective effects without confounding cytotoxicity and are comparable to ranges commonly used in previous in vitro studies investigating huperzine‐mediated neuroprotection [32]. Therefore, the selected doses represent biologically active but nontoxic concentrations suitable for assessing modulation of MPP^+^‐induced oxidative injury. After pretreatment, the culture media with either HupB or E1 were removed; the cultures were then washed once with phosphate‐buffered saline (PBS) and subsequently treated with 1 mM MPP^+^ diluted in the culture media to induce mitochondrial injury in cultures. The cultures were further incubated for 24 h before being subjected to each assay. Since HupB and E1 were dissolved in DMSO, and the final concentration of DMSO did not exceed 0.1% (v/v) in any experimental group, complete culture medium containing 0.1% (v/v) DMSO without HupB, E1, or MPP^+^ was used as the vehicle control.

### 2.5. MTT Cell Viability Assay

The colorimetric MTT assay was performed to evaluate cell viability based on the metabolic activity of mitochondrial oxidoreductase enzymes in the MPP^+^‐treated SH‐SY5Y cultures with or without pretreatment with either HupB or E1. Cells were seeded at a density of 5 × 10^3^ cells/well in a clear 96‐well plate for 24 h before pretreatment with HupB (1, 2, 5, 10, or 20 μM) or E1 (0.1, 0.5, 1, 5, or 10 μg/mL) for 2 h. After 24 h of treatment with 1 mM MPP^+^, the culture medium was replaced with 0.5 mg/mL MTT (M6494; Invitrogen, Waltham, MA, USA) solution dissolved in complete medium and further incubated for 2 h. At the endpoint, the culture medium was removed, and the precipitated purple formazan crystals were completely dissolved in 50 μL DMSO. The absorbance of each culture well was then measured at 570 nm using a microplate reader (Thermo Fisher Scientific, Waltham, MA, USA).

### 2.6. Assessment of Cytotoxicity by LDH Release Assay

Cytotoxicity was further evaluated using the CytoTox 96 Non‐Radioactive Cytotoxicity Assay (G1780, Promega, Madison, WI, USA), according to the manufacturer’s instructions. SH‐SY5Y cells were seeded in clear 96‐well plates at a density of 5 × 10^3^ cells per well and allowed to attach for 24 h. Cells were then pretreated with HupB (1, 2, 5, 10, or 20 μM) or E1 extract (0.1, 0.5, 1, 5, or 10 μg/mL) for 2 h, followed by exposure to 1 mM MPP^+^ for 24 h. After treatment, 50 μL of culture supernatant from each well was transferred to a new 96‐well plate and incubated with the LDH substrate mixture for 30 min at room temperature in the dark. Maximum LDH release was determined by treating positive control wells with lysis buffer provided in the kit 45 min prior to supernatant collection. The enzymatic reaction was stopped by adding the stop solution, and absorbance was measured at 490 nm using a microplate reader (Thermo Fisher Scientific, Waltham, MA, USA). Cytotoxicity was calculated according to the manufacturer’s protocol and expressed as a percentage relative to the maximum LDH release control. All experiments were performed in three independent biological replicates.

### 2.7. Hoechst 33342/Propidium Iodide (PI) Double Staining for Nuclear Staining and Membrane Integrity Assessment

To evaluate nuclear staining patterns and membrane‐compromised cells following MPP^+^ exposure, cultured cells were double‐labeled with Hoechst 33342 and PI. Hoechst 33342 was used to visualize nuclear staining, whereas PI was used to assess cells with compromised plasma membrane integrity. Because the present analysis was based on fluorescence intensity rather than cell‐by‐cell nuclear morphology classification, the assay was interpreted as a relative staining‐intensity assessment rather than as a direct quantitative measure of apoptosis. Cells were seeded at a density of 2.5 × 10^3^ cells/well in a black/clear‐bottom 96‐well plate for 24 h before pretreatment with HupB (2, 5, or 20 μM) or E1 (1, 5, or 10 μg/mL) for 2 h. After 24 h of treatment with MPP^+^, the cells in each well were washed once with PBS, followed by staining with Hoechst 33342 (ab228551; Abcam, Cambridge, UK) diluted in culture medium at a 1:5000 dilution (from the 20 mM stock solution) for 20 min in a humidified incubator in the dark. Each well was then washed once with PBS and stained with PI (P4170; Sigma‐Aldrich) diluted in PBS at a 1:5000 dilution (from the stock solution) for 10 min at room temperature in the dark. Finally, the cells in each well were washed twice with PBS before observation using a Nikon AX R confocal microscopy system (Nikon, Tokyo, Japan). The fluorescence emission intensities for both Hoechst 33342 and PI staining were measured and analyzed using the Nikon NIS‐Element imaging software.

### 2.8. Assessment of Reactive Oxygen Species (ROS) Levels

We postulated that HupB and the E1 extract should be capable of reducing ROS levels in MPP^+^‐treated cells and thereby decrease cell death. The Highly Sensitive DCFH‐DA ROS Assay Kit (R252; Dojindo, Kumamoto, Japan) was used to determine ROS levels. Cells were seeded at a density of 2.5 × 10^3^ cells/well in a black‐bottom 96‐well plate for 24 h before pretreatment with HupB or E1, as previously mentioned in the treatment procedure. After 24 h of treatment with MPP^+^, the cells in each well were washed twice with PBS before being treated with a working solution of highly sensitive DCFH‐DA, according to the manufacturer’s instructions, and incubated for 30 min. Each well was gently washed twice with PBS, and the plate was read using a microplate reader at an excitation wavelength of 490 nm and an emission wavelength of 525 nm. Wells containing DCFH‐DA reagent without cells were included as background controls, and their fluorescence values were subtracted from all experimental readings prior to analysis.

### 2.9. Measurement of GSH Levels and CAT and SOD Activities

We evaluated the cellular antioxidation mechanisms in treated cultures using three parameters: GSH level, CAT enzyme activity, and SOD enzyme activity. Cells were seeded at a density of 5 × 10^5^ cells/well in 6‐well plates, followed by the previously mentioned treatment procedure after seeding for 24 h. At the endpoint, cultures were harvested using cell scrapers and subsequently homogenized in ice‐cold lysis buffer using a sonicator. Protein concentrations in each sample were determined using the Bradford assay. GSH levels and CAT and SOD activities in the lysates were determined using a GSSG/GSH Quantification Kit (G257; Dojindo), a CAT assay kit (CAT‐100; Sigma‐Aldrich), and an SOD assay kit‐WST (S311; Dojindo), respectively, according to the manufacturers’ instructions. Absorbances in each assay were measured using a microplate reader.

### 2.10. Assessment of Mitochondrial Membrane Potential (ΔΨm)

Mitochondrial membrane potential (ΔΨm) was evaluated using the MitoPT JC‐1 Assay Kit (MBS239197; MyBioSource, USA), according to the manufacturer’s instructions with minor modifications. SH‐SY5Y cells were seeded in 6‐well plates at a density of 2 × 10^4^ cells per well and allowed to attach for 24 h. Cells were then pretreated with HupB (2, 5, or 20 μM) or E1 extract (1, 5, or 10 μg/mL) for 2 h, followed by exposure to 1 mM MPP^+^ for 24 h. At the end of treatment, cells were washed once with PBS and incubated with JC‐1 working solution at 37°C for 30 min in the dark. After incubation, cells were washed twice with PBS to remove excess dye. Incubation with 25 μM carbonyl cyanide *m*‐chlorophenylhydrazone (CCCP) for 60 min was performed as a positive control. Fluorescence images were captured using an inverted fluorescence microscope (Zeiss Axio Vert A1, Carl Zeiss, Germany) under identical 1‐s exposure settings for all experimental groups. JC‐1 monomers (green fluorescence) were detected using FITC filter settings, and JC‐1 aggregates (red fluorescence) were detected using TRITC filter settings. Fluorescence intensity was quantified using ImageJ software (NIH, Bethesda, MD, USA). For each condition, at least 3 random fields per well were analyzed across three independent experiments. Background fluorescence was subtracted prior to analysis. Mitochondrial membrane potential was expressed as the ratio of red (aggregated JC‐1) to green (monomeric JC‐1) fluorescence intensity.

### 2.11. Western Blot Analysis of Bax and Bcl‐2 Protein Expression

SH‐SY5Y cells were seeded in 6‐well plates at a density of 1 × 10^5^ cells per well and allowed to attach for 24 h. Cells were then pretreated with HupB (2 or 20 μM) or E1 extract (1 or 10 μg/mL) for 2 h, followed by exposure to 1 mM MPP^+^ for 24 h. At the end of treatment, cells were washed twice with ice‐cold PBS and lysed in radioimmunoprecipitation assay (RIPA) buffer supplemented with 1 mM phenylmethylsulfonyl fluoride (PMSF) as a protease inhibitor. Lysates were collected and centrifuged at 13,000 × g for 10 min at 4°C. The supernatants were collected, and total protein concentrations were determined using the Bradford assay. Protein samples were stored at −80°C until analysis. Equal amounts of protein (30 μg per lane) were separated by SDS–polyacrylamide gel electrophoresis (SDS–PAGE) and transferred onto polyvinylidene fluoride (PVDF) membranes. Membranes were blocked with 5% nonfat dry milk in Tris‐buffered saline containing 0.1% Tween‐20 (TBST) for 1 h at room temperature and incubated overnight at 4°C with the following primary antibodies: rabbit anti‐Bcl‐2 antibody (EPR17509; ab182858; Abcam), rabbit anti‐Bax antibody (E63; ab32503; Abcam), and mouse anti‐β‐actin antibody (MAB1501; MilliporeSigma).

After washing with TBST, membranes were incubated for 1 h at room temperature with horseradish peroxidase (HRP)–conjugated secondary antibodies: anti‐rabbit IgG, HRP‐linked antibody (#7074; Cell Signaling Technology), or anti‐mouse IgG, HRP‐linked antibody (#7076; Cell Signaling Technology). Protein bands were visualized using Immobilon Forte Western HRP substrate (MilliporeSigma) and exposed to medical X‐ray film (Fujifilm, Tokyo, Japan). Band intensities were quantified using ImageJ software (NIH, Bethesda, MD, USA). Bax and Bcl‐2 protein levels were normalized to β‐actin, and the Bax/Bcl‐2 ratio was calculated for each experimental group.

### 2.12. Proteomics Analysis

#### 2.12.1. Proteomics Sample Preparation

Protein extract concentrations in each sample were determined using the Bradford assay. Protein cell pellets were processed using the same method as described previously [37]. Briefly, protein extracts were buffer‐exchanged with 50 mM ammonium bicarbonate buffer using a Bio‐Gel P6 spin column (Bio‐Rad, Hercules, CA, USA). The samples were centrifuged at 14,000 rpm for 5 min, and the supernatant was collected thereafter. Protein concentrations were measured using the Bradford assay and normalized to 1.30 mg/mL protein. The protein solution was reduced with 10 mM dithiothreitol at 65°C for 30 min and alkylated with 25 mM iodoacetamide at room temperature for 20 min in the dark. Proteins were digested with 0.5 μg trypsin at 37°C for 16 h. The reaction was terminated using 10% FA, after which the sample was centrifuged at 14,000 rpm for 10 min. The supernatant was then transferred to a polypropylene vial and subjected to LC–MS/MS analysis.

#### 2.12.2. LC–MS/MS Proteomics Acquisition

Peptide samples were analyzed with the method previously mentioned [37, 38]. Briefly, an Agilent 1290 Infinity II LC system connected to a 6545XT QTOF mass spectrometer (Agilent Technologies, US) and an Agilent AdvanceBio Peptide Mapping column (120 Å, 2.1 × 150 mm, 2.7 μm) were used for the LC–MS proteomics analysis. Mobile phases A and B were 0.1% FA in water and ACN, respectively. Samples were acquired in positive mode. Other LC and MS settings followed previous studies [37, 38].

#### 2.12.3. Proteomics Data Processing

Data were processed as described in a previous publication [38]. Raw Agilent .d files were converted to .mzML and .mzxML files and loaded into MaxQuant software Version 2.4. Trypsin was selected as a digestive enzyme, and two missed cleavages were allowed. Methionine oxidation and N‐terminal acetylation were set as variable modifications, while cysteine carbamidomethylation was a fixed modification. All default parameters for the Agilent QTOF instrument were applied. Protein and peptide match FDR was set at 1.0. LFQ minimum ratio count was reduced to 1 peptide. The human protein database (taxonomy ID: 9606) was acquired from the UniProt website. Proteins identified by site and reverse peptides were removed. LFQ intensity was used for statistical analysis using MetaboAnalyst Version 6.0 (https://www.metaboanalyst.ca/). Data were log_10_‐transformed and mean‐centered. DEPs identified from the statistical analysis were further analyzed using gene ontology (GO) and pathway enrichment analysis with DAVID (https://david.ncifcrf.gov/) [39, 40]. In addition, protein–protein interactions of each DEP were visualized using Cytoscape (https://cytoscape.org/) with the GeneMANIA plugin (https://genemania.org/).

### 2.13. Statistical Analysis

Data are presented as the mean ± standard deviation of three independent experiments. Comparisons were made against the vehicle control or MPP^+^‐treated group, as indicated in each figure legend. Statistical differences between groups were determined using one‐way analysis of variance (ANOVA) with Fisher’s least significant difference post hoc test unless otherwise specified. Differences were considered statistically significant at *p* < 0.05.

## 3. Results

### 3.1. Structural Elucidation of the E1 Extract

Compounds present in the E1 extract were qualitatively analyzed using LC–ESI–QTOF–MS/MS in positive and negative modes. Their structures were inferred from their mass spectra and fragmentation patterns. Table 1 lists the negative and positive molecular ions and the tentative identification of the four compounds detected. The LC–ESI–QTOF–MS/MS total ion chromatograms (TIC) and mass spectra of Huperzine A, Huperzine B, and caffeic acid compounds in *H. carinata* extract are shown in the Supporting Figures 1 and 2, respectively.

**TABLE 1:** Compounds identified in the E1 extracts using LC–ESI–QTOF–MS/MS.

No.	RT^∗^ (min)	m/z	Error (ppm)	Adduct	MS/MS	Tentative identification (MSI level 2)^∗∗^	Molecular formula	Molecular weight (g/mol^−1^)
1	3.542	243.15178	−10.61	[M + H]^+^	243.1490 226.1224 210.0913 134.0962	Huperzine A	C_15_H_18_N_2_O	242.32
	3.515	265.12955	1.70	[M + Na]^+^	265.1304 162.0909 134.0959 70.0651			

2	3.039	257.16489	−19.02	[M + H]^+^	257.1650 240.1622 225.1383 158.0959	Huperzine B	C_16_H_20_N_2_O	256.34

3	3.048	179.03383	3.46	[M − H]^−^	179.0339 135.0444 107.0496 65.3972	Caffeic acid	C_9_H_8_O_4_	180.16

^∗^RT, retention time.

^∗∗^MSI: Metabolomics Standards Initiative.

### 3.2. Huperzine B and the E1 Extract Attenuate MPP^+^‐Associated Reduction in Metabolic Viability Without Significant Changes in LDH Release in SH‐SY5Y Cells

**FIGURE 1:**
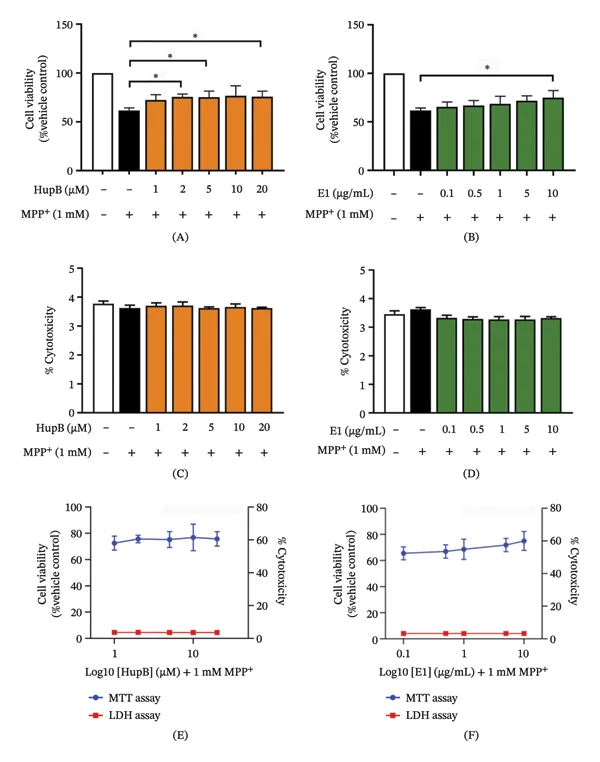
Effects of HupB and E1 on MPP^+^‐associated metabolic viability and LDH release in SH‐SY5Y cells. (A, B) Cell viability assessed by MTT assay following pretreatment with HupB (A) or E1 extract (B) for 2 h prior to 1 mM MPP^+^ exposure for 24 h. (C, D) Lactate dehydrogenase (LDH) release assay measuring membrane integrity under the same treatment conditions. (E, F) Dose–response curves generated from MTT and LDH assays for HupB (E) and E1 (F), respectively. Cells were pretreated with increasing concentrations of HupB (1–20 μM) or E1 (0.1–10 μg/mL) before MPP^+^ exposure. The vehicle control group received complete culture medium containing 0.1% DMSO. Data are presented as mean ± standard deviation (SD) from three independent experiments. Statistical analysis was performed using one‐way ANOVA followed by Fisher’s least significant difference post hoc test. ^∗^ *p* < 0.05 compared with MPP^+^‐treated group.

Cell viability, as determined through measuring mitochondrial enzyme activity via the MTT assay, was significantly decreased (61.7% ± 2.627%) in cells treated with 1 mM MPP^+^ for 24 h compared with the vehicle control group containing 0.1% DMSO. Pretreatment with HupB for 2 h restored cell viability, particularly at concentrations of 2 μM (75.68 ± 2.85%), 5 μM (75.32 ± 6.136%), 10 μM (76.83 ± 10.1%), and 20 μM (75.82 ± 5.568%), all of which showed a significant improvement compared with that of MPP^+^‐treated cells (Figure 1A). However, among the groups pretreated with the E1 extract for 2 h, only the 10 μg/mL concentration significantly increased cell viability (75.03 ± 7.224%) compared with that of the MPP^+^‐treated cells (Figure 1B). These results suggest that HupB at concentrations ≥ 2 μM attenuates the MPP^+^‐associated reduction in metabolic viability in SH‐SY5Y cells, whereas the E1 extract showed a significant effect only at 10 μg/mL. The LDH release assay was performed to assess plasma membrane integrity. Although MPP^+^ significantly reduced MTT‐based metabolic viability, MPP^+^ treatment did not induce a statistically significant increase in extracellular LDH levels compared with the vehicle control under the conditions tested. Similarly, pretreatment with HupB or E1 did not significantly alter LDH release relative to the MPP^+^‐treated group (Figure 1C,D). To further visualize concentration‐dependent effects, dose–response curves were made for both HupB and E1 using the MTT assay and LDH assay (Figure 1E,F). These findings suggest that, under the present exposure condition, MPP^+^ primarily reduced mitochondrial/metabolic activity without producing marked membrane‐disruptive cytotoxicity detectable by LDH release.

### 3.3. Huperzine B and the E1 Extract Alter Hoechst 33342/PI Staining Patterns in MPP^+^‐Treated SH‐SY5Y Cells

**FIGURE 2:**
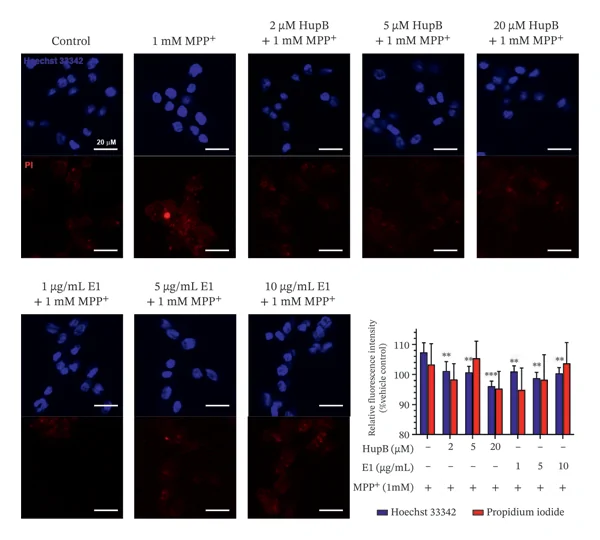
Hoechst 33342 and propidium iodide fluorescence staining in 1‐methyl‐4‐phenylpyridinium (MPP^+^)‐treated SH‐SY5Y cells with and without either HupB or E1 extract pretreatment. The vehicle control group received complete culture medium containing 0.1% DMSO. Scale bar = 20 μm (applies to all panels). The bar graph represents relative Hoechst 33342 and PI fluorescence intensities normalized to the vehicle control group. Because quantification was based on fluorescence intensity rather than direct cell‐by‐cell classification, the data are presented as relative staining intensity and not as a PI/Hoechst ratio or percentage of apoptotic/dead cells. Data are presented as mean ± standard deviation (SD) from three independent experiments. Statistical analysis was performed using one‐way ANOVA followed by Fisher’s least significant difference post hoc test. Double (^∗∗^) and triple asterisks (^∗∗∗^) indicate significant differences at *p* < 0.01 and *p* < 0.001 between the MPP^+^‐treated group and the others that were pretreated with either HupB or E1, respectively.

Hoechst 33342/PI staining was used to evaluate nuclear staining patterns and membrane‐compromised cells following MPP^+^ exposure. In the present analysis, fluorescence intensity was quantified rather than cell‐by‐cell nuclear morphology or PI‐positive nuclei; therefore, Hoechst 33342 fluorescence intensity was not interpreted as a direct measure of early apoptosis. MPP^+^‐treated cells showed a significant increase in relative Hoechst 33342 fluorescence intensity (107.3% ± 3.238% of the vehicle control), whereas PI fluorescence intensity was not significantly different among groups. Pretreatment with HupB at 2 μM (101.1% ± 3.159%), 5 μM (100.7% ± 2.042%), and 20 μM (96.12% ± 1.64%) significantly reduced the MPP^+^‐associated increase in Hoechst 33342 fluorescence intensity. Similarly, pretreatment with E1 extract at 1 μg/mL (101% ± 1.893%), 5 μg/mL (98.77% ± 1.929%), and 10 μg/mL (100.4% ± 1.921%) significantly reduced Hoechst 33342 fluorescence intensity compared with the MPP^+^‐treated group (Figure 2). These findings suggest that HupB and E1 were associated with reduced MPP^+^‐related changes in Hoechst 33,342 nuclear staining intensity; however, because the analysis was based on fluorescence intensity rather than direct cell‐by‐cell classification, these data should not be interpreted as definitive evidence of reduced apoptosis or dead‐cell percentage.

### 3.4. ROS Levels Were Decreased in SH‐SY5Y Cells Pretreated With Huperzine B and the E1 Extract

**FIGURE 3:**
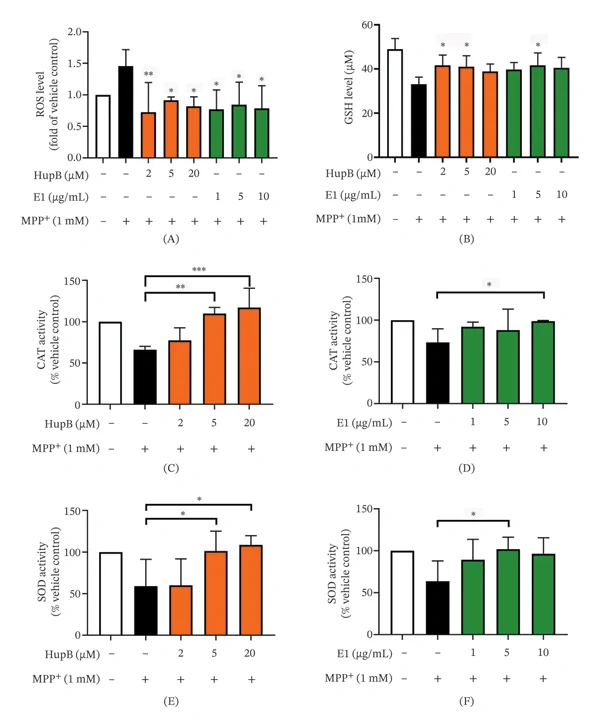
Effects of HupB and E1 on intracellular reactive oxygen species (ROS) levels and antioxidant defense parameters in MPP^+^‐treated SH‐SY5Y cells. (A) Relative intracellular ROS levels measured using the DCFH‐DA assay. (B) Intracellular glutathione (GSH) level expressed in μM. (C, D) Catalase (CAT) activity expressed as a percentage of the vehicle control. (E, F) Superoxide dismutase (SOD) activity expressed as a percentage of the vehicle control. The vehicle control group received complete culture medium containing 0.1% DMSO. Data are presented as mean ± standard deviation (SD) from three independent experiments. Statistical analysis was performed using one‐way ANOVA followed by Fisher’s least significant difference post hoc test. ^∗^ *p* < 0.05, ^∗∗^ *p* < 0.01, and ^∗∗∗^ *p* < 0.001 compared with the MPP^+^‐treated group.

ROS levels, as assessed using the DCFH‐DA assay (Figure 3A), were significantly elevated in cells treated with 1 mM MPP^+^ (1.458 ± 0.2586) compared with those in the vehicle control group, indicating elevation of ROS. Pretreatment with 2, 5, and 20 μM HupB resulted in a significant reduction in ROS levels compared with those in the MPP^+^‐treated group. The 2 μM group exhibited the most pronounced decrease (0.7261 ± 0.4701), followed by the 20 μM (0.8191 ± 0.15) and 5 μM (0.9154 ± 0.05189) groups. These findings suggest that HupB effectively mitigates MPP^+^‐induced oxidative stress, with the strongest antioxidant effect observed at 2 μM. Similarly, pretreatment with the E1 extract at 1, 5, and 10 μg/mL reduced ROS levels to 0.7709 ± 0.3083, 0.8441 ± 0.3561, and 0.7865 ± 0.359, respectively. These findings suggest that both HupB and the E1 extract possess antioxidant properties and could effectively alleviate MPP^+^‐induced oxidative injury.

### 3.5. Intracellular Antioxidation Mechanisms Were Improved in SH‐SY5Y Cells Pretreated With Huperzine B and the E1 Extract

As ROS levels were found to decrease in cells pretreated with either HupB or the E1 extract, we further evaluated the levels of GSH and activities of CAT and SOD enzymes, which are known antioxidant agents within cells (Figure 3B–F). GSH levels were significantly reduced in MPP^+^‐treated cells (33.17 ± 3.129 μM) compared with those in the vehicle control group (48.99 ± 4.817 μM), indicating oxidative stress–induced depletion. Pretreatment with HupB at 2 μM (41.67 ± 4.626 μM) and 5 μM (41.01 ± 5.025 μM) significantly restored GSH levels compared with those in MPP^+^‐treated cells. Among the E1 extract‐treated groups, only the 5 μg/mL concentration (41.67 ± 5.633 μM) significantly elevated GSH levels (Figure 3B). CAT activity was markedly reduced in MPP^+^‐treated cells (66.13 ± 4.004% of the vehicle control); however, pretreatment with HupB at 5 μM (110.0 ± 7.321%) and 20 μM (117.2 ± 23.39%) significantly restored CAT activity compared with that in MPP^+^‐treated cells. For the E1‐pretreated groups, only cells pretreated with 10 μg/mL E1 extract (98.9 ± 0.8095%) showed a significant restoration of CAT activity compared with that in the MPP^+^‐treated group (Figure 3C–D). Lastly, SOD activity was expectedly reduced in MPP^+^‐treated cells (59.17 ± 32.12% of the vehicle control), whereas groups pretreated with HupB at 5 μM (101.4 ± 23.81%) and 20 μM (108.7 ± 11.11%) significantly restored SOD activity compared with that in the MPP^+^‐treated group. Only the group pretreated with 5 μg/mL E1 extract (102.0 ± 14.25%) showed a significant restoration of SOD activity compared with that in the MPP^+^‐treated group (Figure 3E–F). These findings suggest that both HupB and the E1 extract exert protective effects against MPP^+^‐induced ROS accumulation, including the significant restoration of GSH levels and promotion of CAT and SOD activities to help eliminate ROS and cope with the oxidative injury caused by MPP^+^.

### 3.6. Assessment of Mitochondrial Membrane Potential

**FIGURE 4:**
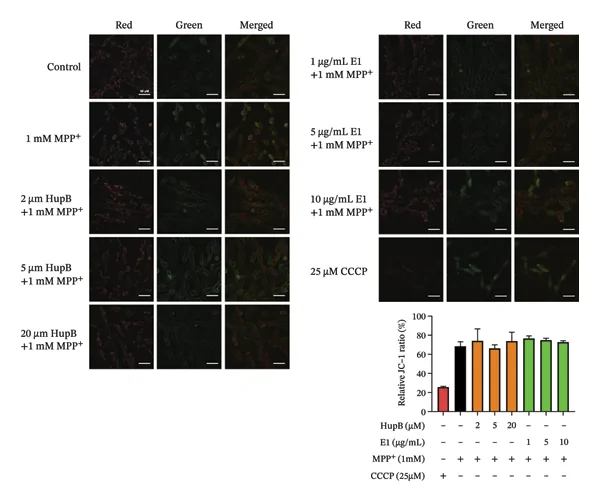
Assessment of mitochondrial membrane potential (ΔΨm) by JC‐1 staining in SH‐SY5Y cells. Representative fluorescence micrographs showing JC‐1 monomers (green), JC‐1 aggregates (red), and merged images for vehicle control, MPP^+^‐treated, HupB‐pretreated, E1‐pretreated, and positive control groups. The vehicle control contained 0.1% DMSO. MPP^+^ treatment reduced the JC‐1 red/green fluorescence ratio, indicating mitochondrial depolarization. Pretreatment with HupB or E1 did not significantly restore the JC‐1 red/green ratio compared with the MPP^+^‐treated group under the present conditions. Cells treated with CCCP (25 μM, 60 min) served as a positive control for mitochondrial depolarization. Scale bar = 50 μm (applies to all panels). Quantitative analysis of mitochondrial membrane potential was performed by calculating the ratio of red fluorescence from JC‐1 aggregates to green fluorescence from JC‐1 monomers using ImageJ software. Data are presented as mean ± standard deviation (SD) from three independent experiments. Statistical analysis was performed using one‐way ANOVA followed by Fisher’s least significant difference post hoc test.

Mitochondrial membrane potential (ΔΨm) was assessed using JC‐1 staining and expressed as the red/green fluorescence intensity ratio. Treatment with 1 mM MPP^+^ for 24 h reduced the JC‐1 red/green ratio to 68.54% ± 4.64% of the vehicle control group, indicating mitochondrial depolarization. However, pretreatment with HupB (2, 5, or 20 μM) or E1 extract (1, 5, or 10 μg/mL) did not result in statistically significant restoration of the JC‐1 ratio compared with the MPP^+^‐treated group under the conditions tested (Figure 4).

### 3.7. Western Blot Analysis of Bax and Bcl‐2 Expression

**FIGURE 5:**
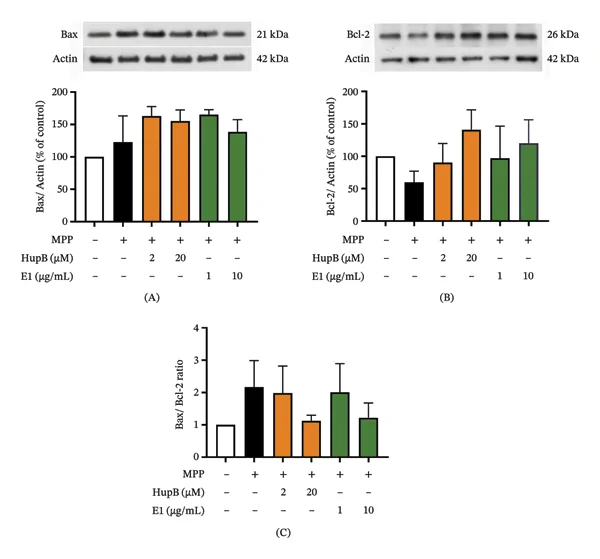
Western blot analysis of Bax and Bcl‐2 expression in SH‐SY5Y cells following MPP^+^ exposure with or without HupB or E1 pretreatment. (A) Representative immunoblot and densitometric analysis of Bax protein expression. (B) Representative immunoblot and densitometric analysis of Bcl‐2 protein expression. (C) Quantification of the Bax/Bcl‐2 ratio calculated from normalized band intensities. SH‐SY5Y cells were pretreated with HupB (2 or 20 μM) or E1 extract (1 or 10 μg/mL) for 2 h prior to 1 mM MPP^+^ exposure for 24 h. Protein expression levels were normalized to β‐actin and expressed relative to the vehicle control group containing 0.1% DMSO. Data are presented as mean ± standard deviation (SD) from three independent experiments. Statistical analysis was performed using one‐way ANOVA followed by Tukey’s post hoc test.

Protein expression levels of Bax and Bcl‐2 were assessed by Western blotting, and the Bax/Bcl‐2 ratio was calculated to evaluate apoptotic signaling (Figure 5). MPP^+^ treatment showed a trend toward increased Bax expression and reduced Bcl‐2 expression compared with control, resulting in an elevated Bax/Bcl‐2 ratio (2.17 ± 0.81). However, these differences did not reach statistical significance under the present experimental conditions. Pretreatment with HupB or E1 did not produce statistically significant changes in Bax or Bcl‐2 expression relative to the MPP^+^‐treated group. Notably, the Bax/Bcl‐2 ratio in cells pretreated with 20 μM HupB (1.12 ± 0.17) and 10 μg/mL E1 (1.22 ± 0.45) showed a trend toward reduction compared with MPP^+^ alone, approaching control levels; however, these changes were not statistically significant.

### 3.8. Proteomics Analysis

A total of 247 protein groups were detected across all experimental groups using LC/MS‐based analysis (Figure 6). Partial least‐squares discriminant analysis (PLS‐DA) revealed distinct clustering among the eight experimental groups tested (Figure 6A–C). Apart from the control group, which received complete medium containing 0.1% DMSO without MPP^+^, HupB, or E1, the other groups were treated with MPP^+^ to induce neurotoxicity. Six groups received pretreatment with either HupB (2, 5, and 20 μM) or the E1 extract (1, 5, and 10 μg/mL), and another group received no pretreatment. A clear separation between the vehicle control and other groups was observed. Three replicates of the control samples were tightly clustered and remained distant from the MPP^+^‐treated samples, reflecting significant proteome alteration upon MPP^+^ exposure. Notably, treatment with either HupB or E1 caused a shift in the proteome profiles away from the MPP^+^ control group in a concentration‐dependent manner. These findings indicate that HupB and E1 pretreatment were associated with changes in the proteomic profiles of MPP^+^‐treated cells. Because these global proteomic findings were not independently validated at the selected protein level, they should be interpreted as exploratory. Comparing the E1 and HupB pretreatment conditions, samples pretreated with the E1 extract showed a clear separation from the MPP^+^‐treated group in the PLS‐DA clustering analysis results, but samples pretreated with HupB overlapped with the MPP^+^‐treated group, implying that the E1 extract caused more notable changes in proteome profiles than the pure HupB compound did. ANOVA and *T*‐test statistical analyses showed that approximately 20% of the total proteins detected were significantly different across the eight experimental groups. A comparison between the control and MPP^+^‐treated groups identified 51 DEPs, with 6 upregulated and 45 downregulated proteins (Figure 6D).

FIGURE 6Proteomic analysis of the untreated, 1‐methyl‐4‐phenylpyridinium (MPP^+^)–treated, and pretreatment groups. (A–C) Partial least‐squares discriminant analysis plots showing separation between the different groups: untreated vs. MPP^+^ (A), MPP^+^ vs. HupB (B), and MPP^+^ vs. E1 (C). (D–F) Volcano plots visualizing fold changes and *p* values measured for the differentially expressed proteins for the same comparisons: untreated vs. MPP^+^ (D), MPP^+^ vs. HupB (E), and MPP^+^ vs. E1 (F). (G–I) Protein interaction networks for each comparison: untreated vs. MPP^+^ (G), MPP^+^ vs. HupB (H), and MPP^+^ vs. E1 (I).

**Figure:**
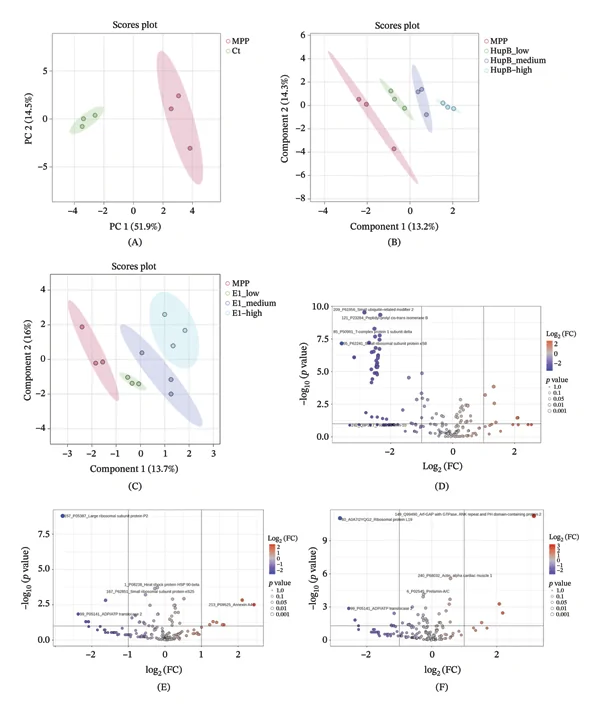

**Figure:**
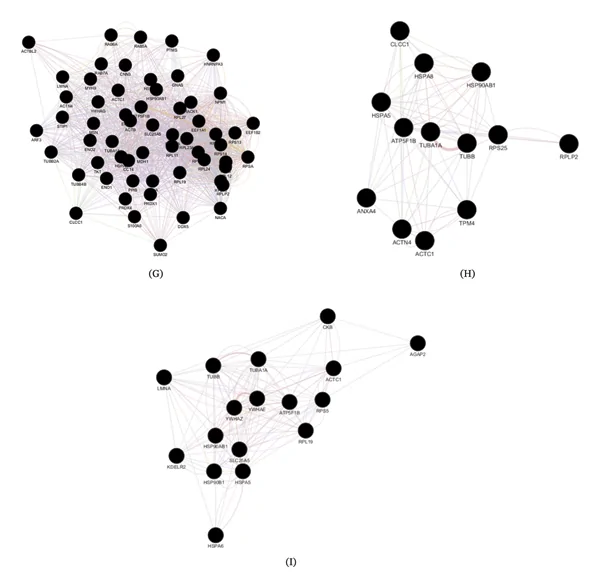

GO enrichment analysis indicated that these proteins were primarily associated with cytoplasmic translation, ribosomal small subunit biogenesis, protein folding, and intracellular protein transport. Interaction network analysis using Cytoscape with the GeneMANIA plugin revealed that most protein interactions occurred through coexpression. Comparisons between the MPP^+^‐treated and HupB‐pretreated groups revealed 13 DEPs (Figure 4E), among which 7 proteins (ACTC1, TUBA1A, HSPA8, ACTN4, CLCC1, RPLP2, and RPS25) showed a recovery pattern similar to the control. Notably, GO enrichment analysis of these DEPs indicated enrichment in biological processes related to the negative regulation of apoptotic processes (Figure 6G). Pretreatment of the cultured cells with E1 extract followed by MPP^+^ resulted in 17 DEPs compared to MPP^+^ treatment alone (Figure 6F). Among these, 7 DEPs were also differentially expressed in the control vs. MPP^+^ comparison. GO analysis of the DEPs altered by E1 cotreatment revealed significant enrichment in negative regulation of apoptotic process, maintenance of protein localization in the endoplasmic reticulum, and protein folding in the endoplasmic reticulum. At the expression level, E1 pretreatment restored the expression of four proteins (RPL19, SLC25A5, ACTC1, and TUBA1A) to levels comparable to the control. These proteins function as components of ribosomes, mitochondrial carrier proteins, and cytoskeletal elements. ACTC1 was annotated in processes related to negative regulation of apoptosis; however, this finding should be interpreted as an exploratory proteomic association rather than direct evidence that E1 protects cells through this pathway. Importantly, ACTC1 and TUBA1A were both restored by pretreatment with either HupB or E1 extract, indicating a shared mechanism. Furthermore, the DEPs identified in the HupB‐ and E1‐pretreated groups primarily interacted through coexpression (Figure 6H‐I). Representative DEPs and their associated biological processes are summarized in Table 2. These results suggest that while HupB is a major component of E1 extract and may contribute to the negative regulation of apoptosis and cytoskeleton‐dependent intracellular transport, the full protective effect of E1 extract likely involves additional compounds or synergistic interactions among multiple bioactive components.

**TABLE 2:** Representative differentially expressed proteins (DEPs) and associated biological processes identified in proteomic comparisons among control, MPP^+^‐treated, HupB‐pretreated, and E1‐pretreated SH‐SY5Y cells.

Comparison	Protein (gene symbol)	Direction vs. MPP^+^	Functional category	Enriched pathway/biological process
Control vs. MPP^+^	Heat shock cognate 71 kDa protein (HSPA8)	↓ in MPP^+^	Molecular chaperone	Protein folding/stress response
	Heat shock protein 90‐beta (HSP90AB1)	↓ in MPP^+^	Chaperone	Proteostasis regulation
	ATP synthase subunit beta (ATP5F1B)	↓ in MPP^+^	Mitochondrial enzyme	Oxidative phosphorylation
	ADP/ATP translocase 2 (SLC25A5)	↓ in MPP^+^	Mitochondrial carrier	Mitochondrial transport
	60S ribosomal protein L19 (RPL19)	↓ in MPP^+^	Ribosomal protein	Cytoplasmic translation
	40S ribosomal protein S25 (RPS25)	↓ in MPP^+^	Ribosomal protein	Ribosome pathway
	Peroxiredoxin‐1 (PRDX1)	↓ in MPP^+^	Antioxidant enzyme	ROS detoxification
	Alpha‐cardiac actin (ACTC1)	↓ in MPP^+^	Cytoskeletal protein	Apoptosis regulation
	Tubulin alpha‐1A chain (TUBA1A)	↓ in MPP^+^	Microtubule component	Cytoskeletal organization

MPP^+^ vs. HupB	78 kDa glucose‐regulated protein (HSPA5)	↑ with HupB	ER chaperone	Protein processing in ER
	Alpha‐cardiac actin (ACTC1)	↑ with HupB	Cytoskeleton	Negative regulation of apoptosis
	Tubulin alpha‐1A chain (TUBA1A)	↑ with HupB	Microtubule	Structural recovery
	ATP synthase subunit beta (ATP5F1B)	↑ with HupB	Mitochondrial enzyme	Bioenergetic restoration
	60S ribosomal protein P2 (RPLP2)	Partial restoration	Ribosome	Translational regulation

MPP^+^ vs. E1	78 kDa glucose‐regulated protein (HSPA5)	↑ with E1	ER stress response	Protein folding
	ADP/ATP translocase 2 (SLC25A5)	↑ with E1	Mitochondrial carrier	Energy metabolism
	60S ribosomal protein L19 (RPL19)	↑ with E1	Ribosomal protein	Translation machinery
	Alpha‐cardiac actin (ACTC1)	↑ with E1	Cytoskeleton	Apoptotic regulation
	14‐3‐3 protein zeta/delta (YWHAZ)	↑ with E1	Signaling adaptor	Cell survival signaling

*Note:* Proteins shown represent key upregulated or downregulated targets identified by LC–MS/MS analysis and selected based on statistical significance and biological relevance. Functional categories and enriched pathways were determined using Gene Ontology (GO) and pathway enrichment analyses. Complete lists of DEPs are provided in Supporting Tables 1–3.

## 4. Discussion

The present study demonstrates that HupB and a *Huperzia carinata* extract (E1) attenuate MPP^+^‐associated reductions in metabolic viability and modulate oxidative stress–associated cellular responses in undifferentiated SH‐SY5Y cells. Because dopaminergic neuronal markers were not assessed, the findings should be interpreted within the context of an MPP^+^‐induced mitochondrial oxidative injury model rather than a fully characterized dopaminergic neuronal model of PD. The present study shows that HupB and E1 extract attenuated MPP^+^‐associated reductions in metabolic viability and modulated oxidative stress–associated responses in SH‐SY5Y cells. These effects were accompanied by reduced ROS accumulation and partial restoration of antioxidant parameters, including GSH levels and CAT/SOD activities. However, because mitochondrial membrane potential and Bax/Bcl‐2 expression were not significantly restored under the present conditions, the data do not support a definitive conclusion that HupB or E1 directly prevents mitochondrial apoptotic signaling. The LC–MS/MS‐based proteomic findings should therefore be interpreted as exploratory and hypothesis‐generating rather than as validated mechanistic evidence. While MPP^+^ is a well‐established mitochondrial Complex I inhibitor and widely employed model for studying oxidative stress–associated dopaminergic injury, it reflects a specific mechanistic aspect of PD pathology. Other toxin‐based models, including rotenone and 6‐OHDA, involve partially overlapping but distinct mechanisms, such as systemic mitochondrial impairment and catecholaminergic oxidative stress. Therefore, validation of HupB and E1 in these complementary systems would help determine whether the observed protective effects extend beyond MPP^+^‐induced mitochondrial dysfunction and represent broader neuroprotective potential. Also, the present study employed a pretreatment paradigm to evaluate protective effects under oxidative stress conditions. While this approach demonstrates preventive potential against mitochondrial toxin exposure, it does not directly assess rescue efficacy after injury initiation. In the context of chronic neurodegenerative disorders such as PD, therapeutic intervention following ongoing neuronal damage represents a clinically relevant scenario. Future studies employing cotreatment and posttreatment regimens will be necessary to determine whether HupB and E1 possess true disease‐modifying or rescue capabilities beyond prophylactic modulation.

LC–ESI–QTOF–MS/MS results revealed that the main bioactive compounds in the E1 extract were HupA and HupB, which are the main constituents found in *H*. *carinata* [41, 42]. Caffeic acid has also been reported in *H*. *serrata* [43, 44] (Table 1). Despite limited evidence on the effects of HupB on improving neuronal viability, our observations are in line with those of previous reports, showing that HupB can protect neuronal cells from oxidative damage under oxygen–glucose deprivation, primarily by decreasing malondialdehyde levels and modulating antioxidant enzyme activity [31, 32]. Although the specific interaction between HupB and MPP^+^ has not been widely characterized, its efficacy in related oxidative injury models supports the premise that HupB acts via similar redox‐regulating pathways. Several studies have investigated the antioxidant effects of HupA, which is a major lycopodium alkaloid extracted from *H*. *serrata* and possesses a chemical structure similar to that of HupB. For example, HupA significantly reduced ROS formation and attenuated caspase‐3 activation in a rat primary cortical neuronal culture treated with amyloid β‐peptide fragment 25–35 [45], suppressed ROS generation, and increased the expression of brain‐derived neurotrophic factor in the murine hippocampal neuronal cell line HT22 [46]. Animal model studies suggest that HupA reduces ROS generation and protects neurons from neuronal death in a kainic acid–induced rat model of epilepsy [47] and a mouse model of repetitive traumatic brain injury [48]. However, as the structure of HupA provides at least four hydrogen‐bond donor and acceptor sites, which facilitates a strong binding affinity with the AChE enzyme and results in potent inhibition, HupA might lead to adverse gastrointestinal and neurological effects due to excess acetylcholine accumulation. In contrast, HupB forms four closed rings with fewer hydrogen‐bonding sites available. Its additional ring may hinder optimal binding, thereby contributing to a lower affinity for AChE than that of HupA [49]. Several early pharmacological studies directly compared HupA and HupB in the same experimental systems. Structural characterization and initial pharmacological evaluation demonstrated that both alkaloids possess anticholinesterase activity, although HupA exhibits higher potency toward AChE inhibition than HupB [50–52]. Comparative in vivo studies further indicated differential effects on skeletal muscle activity, electroencephalographic patterns, and memory enhancement, suggesting distinct pharmacodynamic profiles between the two compounds [53, 54]. These early head‐to‐head evaluations support the notion that HupB displays comparatively weaker cholinergic activity, which may potentially reduce the risk of excessive peripheral cholinergic stimulation. However, direct comparison of HupA and HupB in toxin‐induced dopaminergic stress models has not been extensively investigated and warrants further study. Caffeic acid and its derivatives, such as caffeic acid phenethyl ester (CAPE), have demonstrated neuroprotective activity in both in vivo and in vitro PD models, including 6‐OHDA‐lesioned rats and toxin‐based cellular systems, where they attenuated oxidative stress and apoptotic signaling [55, 56]. These findings support the possibility that phenolic components within E1 may complement alkaloid‐mediated effects. Therefore, the protective effects observed with E1 likely arise from additive or potentially synergistic interactions among multiple bioactive constituents rather than from HupB alone. From a translational perspective, purified compounds such as HupB may offer pharmacokinetic precision, whereas multi‐component extracts may provide broader modulation of cellular stress pathways.

The discrepancy between the MTT and LDH results may reflect differences in the cellular processes measured by these assays. The MTT assay primarily reflects mitochondrial oxidoreductase activity and metabolic viability, whereas LDH release indicates loss of plasma membrane integrity. In the present study, MPP^+^ significantly reduced MTT‐based viability but did not significantly increase extracellular LDH release, suggesting that the selected condition of 1 mM MPP^+^ for 24 h predominantly induced mitochondrial/metabolic impairment rather than extensive membrane‐disruptive cytotoxicity. This interpretation is consistent with the observed ROS elevation and reduction in mitochondrial membrane potential. Therefore, the effects of HupB and E1 should be interpreted mainly as attenuation of MPP^+^‐associated metabolic and oxidative injury rather than prevention of overt necrotic membrane damage.

MPP^+^ is a well‐established inhibitor of mitochondrial Complex I and is known to induce mitochondrial membrane depolarization. In the present study, JC‐1 analysis confirmed that MPP^+^ significantly reduced mitochondrial membrane potential, as evidenced by a decreased red/green fluorescence ratio. However, pretreatment with HupB or E1 did not significantly restore mitochondrial membrane potential under the experimental conditions tested. These findings suggest that although MPP^+^‐induced mitochondrial depolarization was evident, the protective effects of HupB and E1 observed in cell viability and antioxidant assays may not primarily involve direct stabilization of mitochondrial membrane integrity within the 24‐h time frame. It is possible that mitochondrial protective effects, if present, may occur at earlier stages or require more sensitive functional assessments such as direct Complex I activity measurements. It should be noted that mitochondrial membrane potential represents a downstream functional indicator of respiratory chain activity. Direct assessment of Complex I enzymatic activity (e.g., NADH dehydrogenase activity assays) was not performed in the present study. Therefore, while the model employed reflects Complex I–mediated mitochondrial dysfunction, whether HupB or E1 directly modulates Complex I activity remains to be determined. Future studies incorporating specific Complex I activity measurements will help clarify the precise bioenergetic mechanisms underlying the observed protective effects.

The Hoechst 33342/PI staining results should also be interpreted cautiously. In the present study, MPP^+^ exposure increased relative Hoechst 33342 fluorescence intensity, whereas PI fluorescence intensity was not significantly altered among groups. Pretreatment with HupB or E1 reduced the MPP^+^‐associated increase in Hoechst 33342 fluorescence intensity. However, because the analysis was based on fluorescence intensity rather than direct cell‐by‐cell assessment of nuclear condensation, fragmentation, or PI‐positive nuclei, these data should not be interpreted as definitive quantitative evidence of early apoptosis or dead‐cell percentage. Instead, the Hoechst/PI findings provide supportive evidence of MPP^+^‐associated nuclear staining changes that were attenuated by HupB and E1 under the present conditions. More specific apoptosis assays, such as Annexin V/PI flow cytometry, caspase activity assays, or detection of cleaved caspase‐3 and cleaved PARP, would be required to confirm whether apoptotic pathways are directly involved.

The intrinsic apoptotic pathway is frequently associated with mitochondrial dysfunction and alterations in the balance between proapoptotic (Bax) and antiapoptotic (Bcl‐2) proteins. In this study, MPP^+^ exposure showed a tendency to increase the Bax/Bcl‐2 ratio compared with control, consistent with activation of apoptotic signaling; however, these changes exhibited considerable variability and did not reach statistical significance. Similarly, pretreatment with HupB or E1 did not significantly alter Bax or Bcl‐2 expression, although higher concentrations demonstrated a trend toward normalization of the Bax/Bcl‐2 ratio. The observed trends toward normalization of the Bax/Bcl‐2 ratio are consistent with reports from toxin‐based in vivo models, where *Withania somnifera* suppressed intrinsic apoptotic signaling in dopaminergic neurons exposed to paraquat–maneb or MPTP [23]. However, our findings do not provide sufficient evidence to conclude that HupB or E1 directly modulates canonical intrinsic apoptotic signaling under the present experimental conditions. Rather, the Bax/Bcl‐2 data suggest that any apoptosis‐related effect, if present, may be limited, indirect, or dependent on treatment duration and assay sensitivity. Therefore, additional validation using more specific apoptosis markers, such as cleaved caspase‐3, cleaved PARP, Annexin V/PI flow cytometry, or caspase activity assays, will be necessary before firm conclusions can be drawn regarding the involvement of apoptotic pathways in the effects of HupB or E1.

The present findings demonstrate that HupB and E1 attenuate MPP^+^‐induced oxidative stress, as evidenced by reduced DCFH‐DA fluorescence and restoration of intracellular GSH levels and CAT/SOD activities. While DCFH‐DA provides an index of overall intracellular oxidative burden, it does not distinguish specific ROS subtypes or subcellular sources. Given that MPP^+^ primarily impairs mitochondrial Complex I, future studies employing mitochondrial‐targeted probes such as MitoSOX may further clarify the contribution of mitochondrial superoxide to the observed protective effects. Additionally, although antioxidant enzyme activities were restored, the upstream regulatory mechanisms were not directly investigated in the present study. Nuclear factor erythroid 2–related factor 2 (Nrf2) is a central transcriptional regulator of antioxidant response elements and controls expression of genes encoding SOD, CAT, and GSH‐related enzymes. It is therefore plausible that HupB and E1 may influence Nrf2‐mediated signaling pathways under oxidative stress conditions. Future studies examining Nrf2 nuclear translocation, downstream gene expression, or transcriptional activation will be necessary to clarify whether the observed antioxidant enhancement involves direct modulation of this pathway. Furthermore, experiments employing selective inhibitors of CAT or SOD could help determine whether the cytoprotective effects are mechanistically dependent on antioxidant enzyme activity. Therefore, while the current results support a strong association between redox modulation and cellular protection, additional mechanistic studies will be required to establish direct causality. Collectively, the current results support functional redox modulation, while more detailed mechanistic studies remain warranted.

Importantly, the proteomic data indicate that MPP^+^ exposure disrupts ribosomal biogenesis, cytoplasmic translation, and intracellular protein trafficking. These alterations suggest a broader impairment of cellular proteostasis, which has increasingly been recognized as a contributing factor in neurodegenerative disorders. The partial normalization of proteins such as ACTC1 and TUBA1A by both HupB and E1 suggests the stabilization of cytoskeletal and apoptosis‐regulatory processes under redox imbalance conditions, although this interpretation requires targeted validation. Moreover, the more distinct clustering observed in E1‐pretreated samples compared with HupB alone indicates that additional bioactive constituents in the extract may exert synergistic or complementary effects on the cellular proteome. These findings expand on those of previous studies wherein mitochondrial dysfunction was highlighted as a central response to MPP^+^ exposure. For example, quantitative proteomic analysis identified mitochondrial protein dysregulation in MPP^+^‐treated SH‐SY5Y cells, implicating the involvement of organelle‐specific stress responses [57]. Similarly, a meta‐analysis of bioinformatics data emphasized disruptions in ATP synthesis, unfolded protein stress responses, and autophagy in mitochondrial compartments [58]. Recent research in a rat dopaminergic neuronal culture suggested that MPP^+^ could lead to widespread alterations in cytoplasmic protein expression, particularly those involving structural changes, lipid and protein metabolism, and injury‐related proteins [59]. Our data suggest that, in addition to mitochondrial stress, MPP^+^ toxicity significantly alters protein homeostasis at the translational level and intracellular trafficking. The identification of altered protein folding machinery and ribosomal biogenesis components is particularly noteworthy as it points toward a broader proteostatic imbalance that has not been previously emphasized.

Our exploratory proteomic analysis also suggested that HupB and the E1 extract may be associated with changes in apoptosis‐related and stress‐response proteins via the expression of negative regulators of apoptosis and chaperone proteins. Currently, no studies have reported on HupB‐induced alterations in proteomic profiles, but proteomics analysis has shown that HupA downregulates cellular tumor antigen p53 expression in an Alzheimer’s disease model [60]. Other supportive findings from PD‐relevant toxin‐based models include the upregulation of stress‐response proteins and several neuronal processes after cotreatment with Yashtimadhu choorna extract [61], and pretreatment with Ginkgolide B derivatives could protect MPP^+^‐injured MN9D cells via pathways related to the processing of proteins in the endoplasmic reticulum [62]. However, our findings should be interpreted as exploratory and hypothesis‐generating because selected DEPs were not independently validated by targeted molecular approaches. Therefore, the proteomic findings do not establish a definitive mechanism of action for HupB or E1, but rather identify candidate proteins and pathways that may guide future studies. Targeted validation of selected proteins, such as ACTC1, TUBA1A, HSPA8, SLC25A5, or other pathway‐relevant candidates, using Western blotting, qPCR, enzyme activity assays, or functional perturbation approaches will be necessary before firm conclusions can be drawn regarding their roles in HupB‐ or E1‐associated cytoprotection.

Several limitations of this study should be acknowledged. First, experiments were performed using undifferentiated SH‐SY5Y neuroblastoma cells, which, although widely used in MPP^+^ toxicity studies [63–66], do not fully exhibit the phenotype of mature dopaminergic neurons. Differentiation of SH‐SY5Y cells or the use of primary dopaminergic neuronal cultures may provide enhanced physiological relevance through increased expression of dopaminergic markers such as TH and DAT. The present study prioritized a reproducible mitochondrial oxidative stress model and did not directly quantify TH or DAT expression. Therefore, while the MPP^+^ paradigm effectively reproduces Complex I–mediated oxidative and proteostatic disturbances [35], it does not encompass all dopaminergic neuron‐specific molecular features. Future studies employing differentiated SH‐SY5Y cells, primary neuronal cultures, or in vivo PD models would further strengthen translational relevance. Second, although global proteomic analysis identified DEPs associated with apoptotic regulation, cytoskeletal organization, and protein folding, these proteomic findings were not independently validated by targeted Western blotting, qPCR, enzyme activity assays, or functional perturbation experiments. Therefore, the proteomics data should be interpreted as exploratory and hypothesis‐generating rather than as definitive evidence of mechanism. In particular, α‐synuclein expression or aggregation was not directly evaluated. While α‐synuclein accumulation represents a central feature of PD, the acute MPP^+^ exposure model primarily induces mitochondrial Complex I inhibition and oxidative stress rather than robust synucleinopathy. Therefore, the present findings reflect mitochondrial toxin–mediated neuronal stress relevant to PD but do not fully recapitulate α‐synuclein–associated pathology. Future studies using chronic or genetic PD models will be required to determine whether HupB or *H. carinata* extract influences synuclein‐related mechanisms. Third, phytochemical characterization of the *H. carinata* extract focused primarily on major detectable constituents; therefore, comprehensive and quantitative profiling of additional bioactive compounds remains warranted. Finally, the present findings are based on an in vitro cellular model and therefore cannot fully capture the complex multicellular and systemic processes involved in PD pathogenesis. Although the MPP^+^‐induced SH‐SY5Y model provides a useful platform for investigating mitochondrial dysfunction and oxidative stress mechanisms, it does not reflect factors such as neuroinflammation, neuronal network interactions, pharmacokinetics, or blood–brain barrier penetration. Consequently, the translational relevance of HupB and *H. carinata* extract requires further investigation in appropriate in vivo PD models to determine whether the protective effects observed in vitro extend to physiological systems.

## 5. Conclusion

In summary, HupB and the E1 extract derived from *H. carinata* attenuated MPP^+^‐associated reductions in metabolic viability and oxidative stress parameters in an SH‐SY5Y toxin‐based oxidative injury model. These effects were associated with reduced ROS accumulation and partial restoration of antioxidant defenses, including GSH levels and CAT/SOD activities. However, HupB and E1 did not significantly restore mitochondrial membrane potential or significantly alter Bax/Bcl‐2 expression under the present conditions. Proteomic analysis suggested changes in proteins related to apoptosis regulation, cytoskeletal organization, and cellular stress responses, but these findings require independent validation. Therefore, the present study supports further investigation of HupB and *H. carinata* extract as redox‐modulating candidates rather than establishing a definitive neuroprotective mechanism. Although this study does not recapitulate the full molecular complexity of PD, the findings provide mechanistic insight into cellular stress modulation and warrant further evaluation in more physiologically relevant neuronal and in vivo models.

## Author Contributions

Sakan Warinhomhoun and Kittikun Viwatpinyo: conceptualization. Sakan Warinhomhoun, Kittikun Viwatpinyo, Poommaree Namchaiw, Pipob Suwanchaikasem, Sudichhya Tamrakar, and Sujira Mukda: methodology, investigation, and software. Sakan Warinhomhoun, Kittikun Viwatpinyo, Poommaree Namchaiw, Pipob Suwanchaikasem, and Amit Jaisi: validation, formal analysis, and resources. Sakan Warinhomhoun, Kittikun Viwatpinyo, Poommaree Namchaiw, and Pipob Suwanchaikasem: writing–original draft and writing–review and editing. Sakan Warinhomhoun and Kittikun Viwatpinyo: supervision. Sakan Warinhomhoun and Kittikun Viwatpinyo: visualization, project administration, and funding acquisition.

## Funding

This research was supported by Thailand Science Research and Innovation Fund Contract No. FF‐WU67‐10.

## Disclosure

All authors have read and agreed to the published version of the manuscript.

## Conflicts of Interest

The authors declare no conflicts of interest.

## Supporting Information

Additional supporting information can be found online in the Supporting Information section.

## Supporting information


**Supporting Information** The supporting file contains Supporting Tables 1–3, which present the full list of differentially expressed proteins identified in this study, including protein ID, protein and gene names, fold changes in terms of log_10_ (P), corresponding *p* values, and false discovery rate (FDR). Supporting Figure 1 shows the LC–ESI–QTOF–MS/MS total ion chromatograms (TICs) of the main identified compounds in the *H. carinata* extract, including Huperzine A, Huperzine B, and caffeic acid. Supporting Figure 2 presents the corresponding mass spectra of these identified compounds, supporting their tentative identification in the extract.

## Data Availability

The data that support the findings of this study are available from the corresponding author upon reasonable request.
