# Supplementary material for: Neuroprotective Effects of Huperzine B and Huperzia carinata Extract Against MPP+‐Induced Oxidative Stress in SH‐SY5Y Cells
Source: Adv Pharmacol Pharm Sci. 2026 Sep 21;2026:6446024. doi: 10.1155/adpp/6446024 (PMC13591997; doi:10.1155/adpp/6446024)
Supplement: Supplementary file 1 [file ADPP-2026-6446024-s001.zip › Supplementary material LC-MS.docx]

**Supplementary material**

(A)

(B)

(C)

**Supplementary Figure 1.** LC-ESI-QTOF-MS/MS total ion chromatogram (TIC) of (**A**) huperzine A, (**B**) huperzine B, and (**C**) caffeic acid compounds in *H. carinata* extract.

(A)

(B)

(C)

**Supplementary Figure 2**. Mass spectrum of the main identified (**A**) huperzine A, (**B**) huperzine B, and (**C**) caffeic acid compounds in *H. carinata* extract.
