# Supplementary material for: Neuroprotective Effects of Huperzine B and Huperzia carinata Extract Against MPP+‐Induced Oxidative Stress in SH‐SY5Y Cells
Source: Adv Pharmacol Pharm Sci. 2026 Sep 21;2026:6446024. doi: 10.1155/adpp/6446024 (PMC13591997; doi:10.1155/adpp/6446024)
Supplement: Supplementary file 1 [file ADPP-2026-6446024-s001.zip › Supplementary Table.docx]

**Supplementary Table 1**: Statistical analysis of proteomic data comparing MPP^+^-treated and control groups. List of significantly altered proteins identified by LC-MS/MS analysis, including fold changes in terms of log10(P), p-values, and false discovery rate (FDR). Statistical significance was determined using Student’s t-test.

| Selected Protein ID | Protein name | Gene name | Number of proteins | Razor + unique peptides | Sequence coverage [%] | Score | t.stat | p.value | LOG10(P) | FDR |
| --- | --- | --- | --- | --- | --- | --- | --- | --- | --- | --- |
| P08238 | Heat shock protein HSP 90-beta | HSP90AB1 | 9 | 24 | 39.9 | 208.14 | -3.9495 | 0.016825 | 1.774 | 0.064126 |
| P60709 | Actin, cytoplasmic 1 | ACTB | 93 | 23 | 82.9 | 323.31 | 3.355 | 0.028435 | 1.5461 | 0.097354 |
| P11142 | Heat shock cognate 71 kDa protein | HSPA8 | 38 | 21 | 38.9 | 150.09 | -7.9068 | 0.0013842 | 2.8588 | 0.0071081 |
| P02545 | Prelamin-A/C | LMNA | 24 | 19 | 29.1 | 56.619 | 3.7952 | 0.019183 | 1.7171 | 0.07176 |
| P35579 | Myosin-9 | MYH9 | 14 | 17 | 9.7 | 43.123 | -10.498 | 0.00046543 | 3.3321 | 0.0026116 |
| O43707 | Alpha-actinin-4 | ACTN4 | 53 | 15 | 22.6 | 41.397 | 3.5008 | 0.024879 | 1.6042 | 0.08974 |
| P06576 | ATP synthase subunit beta, mitochondrial | ATP5F1B | 7 | 14 | 36.9 | 84.083 | -7.8208 | 0.0014429 | 2.8408 | 0.007109 |
| P68104 | Elongation factor 1-alpha 1 | EEF1A1 | 82 | 13 | 36.4 | 151.31 | 4.8088 | 0.008593 | 2.0659 | 0.035393 |
| P00338 | L-lactate dehydrogenase A chain | LDHA | 9 | 11 | 38.3 | 91.416 | 3.452 | 0.026006 | 1.5849 | 0.092163 |
| P10809 | 60 kDa heat shock protein, mitochondrial | HSPD1 | 22 | 10 | 21.8 | 47.631 | 5.7534 | 0.0045256 | 2.3443 | 0.019558 |
| P06733 | Alpha-enolase | ENO1 | 18 | 10 | 29.7 | 71.494 | -3.5331 | 0.024163 | 1.6168 | 0.088745 |
| Q06830 | Peroxiredoxin-1 | PRDX1 | 8 | 7 | 33.2 | 22.662 | -8.377 | 0.0011108 | 2.9544 | 0.0059047 |
| P26038 | Moesin | MSN | 15 | 7 | 10.6 | 14.095 | 5.2861 | 0.0061447 | 2.2115 | 0.025859 |
| P63244 | Small ribosomal subunit protein RACK1 | RACK1 | 21 | 5 | 16.4 | 11.131 | -137.45 | 1.68E-08 | 7.7746 | 8.32E-07 |
| P61981 | 14-3-3 protein gamma | YWHAG | 11 | 5 | 35.2 | 11.177 | -3.3684 | 0.028087 | 1.5515 | 0.097354 |
| P62241 | Small ribosomal subunit protein eS8 | RPS8 | 4 | 5 | 26.4 | 12.487 | -96.324 | 6.96E-08 | 7.1571 | 1.76E-06 |
| P68371 | Tubulin beta-4B chain | TUBB4B | 23 | 5 | 57.8 | 88.376 | -4.7823 | 0.0087607 | 2.0575 | 0.035393 |
| P08865 | Small ribosomal subunit protein uS2 | RPSA | 9 | 4 | 16.6 | 48.73 | -4.1736 | 0.01399 | 1.8542 | 0.054347 |
| P06748 | Nucleophosmin | NPM1 | 19 | 4 | 17 | 11.611 | 6.0654 | 0.003731 | 2.4282 | 0.017364 |
| P62277 | Small ribosomal subunit protein uS15 | RPS13 | 3 | 4 | 27.2 | 4.9846 | -130.62 | 2.06E-08 | 7.6861 | 8.32E-07 |
| A0A7I2YQG2 | Ribosomal protein L19 | RPL19 | 6 | 3 | 19.9 | 3.7326 | -40.868 | 2.14E-06 | 5.6691 | 2.28E-05 |
| P50991 | T-complex protein 1 subunit delta | CCT4 | 5 | 3 | 5.2 | 6.2735 | -184.91 | 5.13E-09 | 8.2898 | 3.46E-07 |
| P62750 | Large ribosomal subunit protein uL23 | RPL23A | 7 | 3 | 19.9 | 3.1012 | -17.114 | 6.84E-05 | 4.1651 | 0.00040622 |
| P05141 | ADP/ATP translocase 2 | SLC25A5 | 2 | 3 | 10.4 | 6.7728 | -65.603 | 3.23E-07 | 6.4902 | 5.44E-06 |
| P62913 | Large ribosomal subunit protein uL5 | RPL11 | 4 | 3 | 16.9 | 5.448 | -113.83 | 3.57E-08 | 7.4472 | 1.20E-06 |
| Q07020 | Large ribosomal subunit protein eL18 | RPL18 | 9 | 2 | 12.8 | 8.385 | -35.476 | 3.77E-06 | 5.4239 | 3.46E-05 |
| P29401 | Transketolase | TKT | 10 | 2 | 2.9 | 3.3454 | 6.043 | 0.0037822 | 2.4223 | 0.017364 |
| P62263 | Small ribosomal subunit protein uS11 | RPS14 | 3 | 2 | 15.9 | 6.0594 | 4.3757 | 0.011915 | 1.9239 | 0.047193 |
| P40429 | Large ribosomal subunit protein uL13 | RPL13A | 11 | 2 | 9.4 | 2.49 | -37.952 | 2.88E-06 | 5.5408 | 2.80E-05 |
| P40925 | Malate dehydrogenase, cytoplasmic | MDH1 | 7 | 2 | 8.7 | 11.502 | -50.445 | 9.24E-07 | 6.0343 | 1.10E-05 |
| Q96S66 | Chloride channel CLIC-like protein 1 | CLCC1 | 18 | 2 | 2.9 | 2.1878 | 5.847 | 0.0042671 | 2.3699 | 0.019155 |
| P23284 | Peptidyl-prolyl cis-trans isomerase B | PPIB | 5 | 2 | 12 | 8.3719 | -340.98 | 4.44E-10 | 9.3528 | 4.48E-08 |
| P61204 | ADP-ribosylation factor 3 | ARF3 | 23 | 2 | 16 | 5.5699 | -52.261 | 8.02E-07 | 6.0956 | 1.01E-05 |
| P24534 | Elongation factor 1-beta | EEF1B2 | 2 | 2 | 9.8 | 4.3995 | -30.667 | 6.74E-06 | 5.1716 | 5.23E-05 |
| G3V279 | Enhancer of rudimentary homolog | ERH | 2 | 2 | 57.7 | 3.1801 | -33.694 | 4.63E-06 | 5.3346 | 3.74E-05 |
| P35268 | Large ribosomal subunit protein eL22 | RPL22 | 8 | 2 | 18.8 | 4.1487 | -34.439 | 4.24E-06 | 5.3725 | 3.73E-05 |
| P05387 | Large ribosomal subunit protein P2 | RPLP2 | 2 | 2 | 35.7 | 5.7016 | -45.034 | 1.45E-06 | 5.8374 | 1.63E-05 |
| P06703 | Protein S100-A6 | S100A6 | 3 | 2 | 27.8 | 22.514 | 7.8721 | 0.0014076 | 2.8515 | 0.0071081 |
| P09104 | Gamma-enolase | ENO2 | 5 | 2 | 19.1 | 6.5022 | -25.728 | 1.36E-05 | 4.8678 | 9.13E-05 |
| P30050 | Large ribosomal subunit protein uL11 | RPL12 | 3 | 2 | 15.2 | 3.3219 | -75.856 | 1.81E-07 | 6.7423 | 3.66E-06 |
| P62851 | Small ribosomal subunit protein eS25 | RPS25 | 1 | 2 | 15.2 | 4.4957 | -21.289 | 2.88E-05 | 4.5408 | 0.00018171 |
| Q15417 | Calponin-3 | CNN3 | 14 | 2 | 7.6 | 3.3488 | -79.308 | 1.52E-07 | 6.8196 | 3.40E-06 |
| P20340 | Ras-related protein Rab-6A | RAB6A | 11 | 1 | 5.3 | 2.3989 | -33.688 | 4.63E-06 | 5.3343 | 3.74E-05 |
| P63092 | Guanine nucleotide-binding protein G(s) subunit alpha isoforms short | GNAS | 33 | 1 | 2.8 | 2.2429 | -26.134 | 1.27E-05 | 4.8949 | 9.13E-05 |
| P20339 | Ras-related protein Rab-5A | RAB5A | 15 | 1 | 5.1 | 2.227 | -25.838 | 1.33E-05 | 4.8752 | 9.13E-05 |
| P17844 | Probable ATP-dependent RNA helicase DDX5 | DDX5 | 17 | 1 | 2 | 2.993 | -57.154 | 5.61E-07 | 6.2509 | 8.39E-06 |
| P20962 | Parathymosin | PTMS | 4 | 1 | 10.8 | 2.1448 | -99.494 | 6.12E-08 | 7.2133 | 1.76E-06 |
| P51149 | Ras-related protein Rab-7a | RAB7A | 11 | 1 | 5.3 | 2.189 | -24.399 | 1.67E-05 | 4.7762 | 0.0001091 |
| P51991 | Heterogeneous nuclear ribonucleoprotein A3 | HNRNPA3 | 10 | 1 | 2.6 | 3.837 | -19.963 | 3.72E-05 | 4.43 | 0.00022745 |
| E9PAV3 | Nascent polypeptide-associated complex subunit alpha, muscle-specific form | NACA | 9 | 1 | 0.7 | 7.9787 | -56.646 | 5.82E-07 | 6.2354 | 8.39E-06 |
| P83731 | Large ribosomal subunit protein eL24 | RPL24 | 5 | 1 | 8.3 | 2.7992 | -26.023 | 1.30E-05 | 4.8875 | 9.13E-05 |
| P31948 | Stress-induced-phosphoprotein 1 | STIP1 | 3 | 1 | 2.4 | 2.1671 | -54.419 | 6.83E-07 | 6.1658 | 9.19E-06 |
| P61956 | Small ubiquitin-related modifier 2 | SUMO2 | 5 | 1 | 12.6 | 3.3221 | -379.24 | 2.90E-10 | 9.5375 | 4.48E-08 |
| P61353 | Large ribosomal subunit protein eL27 | RPL27 | 4 | 1 | 6.6 | 2.2576 | -37.844 | 2.91E-06 | 5.5358 | 2.80E-05 |
| Q13162 | Peroxiredoxin-4 | PRDX4 | 3 | 1 | 7 | 3.471 | -5.7447 | 0.0045506 | 2.3419 | 0.019558 |
| P68032 | Actin, alpha cardiac muscle 1 | ACTC1 | 19 | 1 | 36.3 | 32.63 | 6.5948 | 0.0027386 | 2.5625 | 0.013172 |
| Q13885 | Tubulin beta-2A chain | TUBB2A | 4 | 1 | 58 | 2.5019 | -67.611 | 2.87E-07 | 6.5425 | 5.27E-06 |
| Q562R1 | Beta-actin-like protein 2 | ACTBL2 | 1 | 1 | 22.3 | 34.921 | 14.255 | 0.00014067 | 3.8518 | 0.00081188 |
| Q71U36 | Tubulin alpha-1A chain | TUBA1A | 4 | 1 | 41.5 | 10.528 | 9.7485 | 0.0006202 | 3.2075 | 0.0033859 |

**Supplementary Table 2**: Statistical analysis of proteomic data comparing MPP^+^-treated and HupB-pretreated groups. List of significantly altered proteins identified by LC-MS/MS analysis, including fold changes in terms of log10(P), p-values, and false discovery rate (FDR). Statistical significance was determined using Student’s t-test.

| Selected Protein ID | Protein name | Gene name | Number of proteins | Razor + unique peptides | Sequence coverage [%] | Score | t.stat | p.value | LOG10(p) | FDR |
| --- | --- | --- | --- | --- | --- | --- | --- | --- | --- | --- |
| P08238 | Heat shock protein HSP 90-beta | HSP90AB1 | 9 | 24 | 208.1 | 39.9 | -5.7388 | 0.00018802 | 3.7258 | 0.014275 |
| P11142 | Heat shock cognate 71 kDa protein | HSPA8 | 38 | 21 | 150.1 | 38.9 | -3.8092 | 0.0034334 | 2.4643 | 0.055286 |
| P07437 | Tubulin beta chain | TUBB | 20 | 20 | 323.3 | 60.1 | 4.4404 | 0.0012536 | 2.9018 | 0.036075 |
| O43707 | Alpha-actinin-4 | ACTN4 | 53 | 15 | 41.4 | 22.6 | 3.799 | 0.0034917 | 2.457 | 0.055286 |
| P11021 | Endoplasmic reticulum chaperone BiP | HSPA5 | 5 | 15 | 52.0 | 26.8 | -4.9182 | 0.00060645 | 3.2172 | 0.028806 |
| P06576 | ATP synthase subunit beta, mitochondrial | ATP5F1B | 7 | 14 | 84.1 | 36.9 | 3.5278 | 0.0054665 | 2.2623 | 0.079895 |
| A0A2R8YE05 | Tropomyosin alpha-4 chain | TPM4 | 8 | 2 | 3.4 | 26.5 | -4.3175 | 0.001519 | 2.8185 | 0.036075 |
| Q96S66 | Chloride channel CLIC-like protein 1 | CLCC1 | 18 | 2 | 2.2 | 2.9 | 4.3408 | 0.0014644 | 2.8343 | 0.036075 |
| P05387 | Large ribosomal subunit protein P2 | RPLP2 | 2 | 2 | 5.7 | 35.7 | -20.62 | 1.59E-09 | 8.798 | 3.03E-07 |
| P62851 | Small ribosomal subunit protein eS25 | RPS25 | 1 | 2 | 4.5 | 15.2 | -5.6073 | 0.0002254 | 3.6471 | 0.014275 |
| P09525 | Annexin A4 | ANXA4 | 7 | 1 | 2.3 | 5.0 | 3.8713 | 0.0031025 | 2.5083 | 0.055286 |
| P68032 | Actin, alpha cardiac muscle 1 | ACTC1 | 19 | 1 | 32.6 | 36.3 | 3.823 | 0.0033573 | 2.474 | 0.055286 |
| Q71U36 | Tubulin alpha-1A chain | TUBA1A | 4 | 1 | 10.5 | 41.5 | 4.4857 | 0.0011686 | 2.9323 | 0.036075 |

**Supplementary Table 3**: Statistical analysis of proteomic data comparing MPP^+^-treated and E1-pretreated groups. List of significantly altered proteins identified by LC-MS/MS analysis, including fold changes in terms of log10(P), p-values, and false discovery rate (FDR). Statistical significance was determined using Student’s t-test.

| Selected Protein ID | Protein name | Gene name | Number of proteins | Razor + unique peptides | Sequence coverage [%] | Score | t.stat | p.value | LOG10(p) | FDR |
| --- | --- | --- | --- | --- | --- | --- | --- | --- | --- | --- |
| P08238 | Heat shock protein HSP 90-beta | HSP90AB1 | 9 | 24 | 208.1 | 39.9 | 4.4367 | 0.001261 | 2.8993 | 0.022858 |
| P07437 | Tubulin beta chain | TUBB | 20 | 20 | 323.3 | 60.1 | -3.6823 | 0.0042299 | 2.3737 | 0.052169 |
| P02545 | Prelamin-A/C | LMNA | 24 | 19 | 56.6 | 29.1 | 6.478 | 7.09E-05 | 4.1493 | 0.0032794 |
| P14625 | Endoplasmin | HSP90B1 | 22 | 16 | 34.9 | 22.2 | 3.5881 | 0.0049444 | 2.3059 | 0.057169 |
| P11021 | Endoplasmic reticulum chaperone BiP | HSPA5 | 5 | 15 | 52.0 | 26.8 | 4.5772 | 0.0010149 | 2.9936 | 0.020862 |
| P06576 | ATP synthase subunit beta, mitochondrial | ATP5F1B | 7 | 14 | 84.1 | 36.9 | -4.969 | 0.00056248 | 3.2499 | 0.013007 |
| P63104 | 14-3-3 protein zeta/delta | YWHAZ | 13 | 12 | 74.7 | 55.9 | 3.5373 | 0.0053807 | 2.2692 | 0.058555 |
| P12277 | Creatine kinase B-type | CKB | 13 | 8 | 36.8 | 29.9 | -4.0671 | 0.0022608 | 2.6457 | 0.034853 |
| P62258 | 14-3-3 protein epsilon | YWHAE | 12 | 8 | 24.0 | 45.5 | 3.1914 | 0.0096321 | 2.0163 | 0.098996 |
| A0A7I2YQG2 | Ribosomal protein L19 | RPL19 | 6 | 3 | 3.7 | 19.9 | 34.694 | 9.38E-12 | 11.028 | 8.68E-10 |
| P05141 | ADP/ATP translocase 2 | SLC25A5 | 2 | 3 | 6.8 | 10.4 | 4.3886 | 0.0013591 | 2.8667 | 0.022858 |
| Q99490 | Arf-GAP with GTPase, ANK repeat and PH domain-containing protein 2 | AGAP2 | 3 | 2 | 1.9 | 1.2 | -36.417 | 5.80E-12 | 11.237 | 8.68E-10 |
| P46782 | Small ribosomal subunit protein uS7 | RPS5 | 5 | 2 | 18.7 | 18.1 | -3.7974 | 0.0035007 | 2.4558 | 0.049818 |
| P17066 | Heat shock 70 kDa protein 6 | HSPA6 | 6 | 1 | 49.2 | 6.2 | 6.0756 | 0.00011948 | 3.9227 | 0.0044208 |
| P33947 | ER lumen protein-retaining receptor 2 | KDELR2 | 2 | 1 | 11.3 | 9.4 | -5.0062 | 0.00053246 | 3.2737 | 0.013007 |
| P68032 | Actin, alpha cardiac muscle 1 | ACTC1 | 19 | 1 | 32.6 | 36.3 | -9.4629 | 2.63E-06 | 5.58 | 0.00016219 |
| Q71U36 | Tubulin alpha-1A chain | TUBA1A | 4 | 1 | 10.5 | 41.5 | -5.6845 | 0.00020259 | 3.6934 | 0.0062467 |
